# Targeted disruption of a single sex pheromone receptor gene completely abolishes *in vivo* pheromone response in the silkmoth

**DOI:** 10.1038/srep11001

**Published:** 2015-06-05

**Authors:** Takeshi Sakurai, Hidefumi Mitsuno, Akihisa Mikami, Keiro Uchino, Masashi Tabuchi, Feng Zhang, Hideki Sezutsu, Ryohei Kanzaki

**Affiliations:** 1 Research Center for Advanced Science and Technology, The University of Tokyo, 4-6-1 Komaba, Meguro-ku, Tokyo 153-8904, Japan; 2 Transgenic Silkworm Research Unit, National Institute of Agrobiological Sciences, 1-2 Owashi, Tsukuba, Ibaraki 305-8634, Japan; 3 McGovern Institute for Brain Research, Massachusetts Institute of Technology, Cambridge, MA 02139, USA

## Abstract

Male moths use species-specific sex pheromones to identify and orientate toward conspecific females. Odorant receptors (ORs) for sex pheromone substances have been identified as sex pheromone receptors in various moth species. However, direct *in vivo* evidence linking the functional role of these ORs with behavioural responses is lacking. In the silkmoth, *Bombyx mori*, female moths emit two sex pheromone components, bombykol and bombykal, but only bombykol elicits sexual behaviour in male moths. A sex pheromone receptor BmOR1 is specifically tuned to bombykol and is expressed in specialized olfactory receptor neurons (ORNs) in the pheromone sensitive long sensilla trichodea of male silkmoth antennae. Here, we show that disruption of the *BmOR1* gene, mediated by transcription activator-like effector nucleases (TALENs), completely removes ORN sensitivity to bombykol and corresponding pheromone-source searching behaviour in male moths. Furthermore, transgenic rescue of BmOR1 restored normal behavioural responses to bombykol. Our results demonstrate that BmOR1 is required for the physiological and behavioural response to bombykol, demonstrating that it is the receptor that mediates sex pheromone responses in male silkmoths. This study provides the first direct evidence that a member of the sex pheromone receptor family in moth species mediates conspecific sex pheromone information for sexual behaviour.

In a wide range of organisms, a unique class of chemicals called sex pheromones plays important roles in intraspecific communication between individuals of the opposite sex. In most moth species, males largely depend on species-specific sex pheromones emitted by females to identify and orient towards an appropriate mating partner among a large number of sympatric insect species^1^,^2^. To successfully locate a mating partner, male moths have evolved a sophisticated olfactory system to detect and discriminate conspecific pheromones from similar compounds that are often emitted from females of other moth species. This high selectivity of the olfactory system has resulted in moth sex pheromone perception becoming model system for understanding the molecular and neural mechanisms that underlie chemical signal-mediated mate recognition.

In insects, odorants are detected by the heteromeric odorant-gated ion channel complex, which is formed by an odorant receptor (OR) and a co-receptor (Orco, formerly called Or83b) that are expressed on the dendritic membrane of olfactory receptor neurons (ORNs)^3^,^4^,^5^. The odorant response profile of this complex is determined by the odorant receptor with both units contributing to the ion channel properties of the complex^6^. To date, ORs for sex pheromone substances have been identified as sex pheromone receptors in various moth species^7^,^8^,^9^,^10^,^11^,^12^,^13^,^14^,^15^,^16^,^17^,^18^,^19^. These receptors have been characterized based on observations showing that ectopic expression confers heterologous cell responsiveness to pheromone component(s) in corresponding species, male antennae-specific or -biased expression patterns, histological localization in ORNs in pheromone sensitive olfactory sensilla, and amino acid sequence similarity among species. Thus, these ORs are hypothesized to play a central role in the detection and discrimination of pheromones, and to mediate pheromone information for behavioural responses in male moths. However, direct *in vivo* evidence linking the functional role of these ORs with behavioural responses is lacking; consequently, it remains unclear whether these ORs are responsible for pheromone responses in *in vivo*.

If sex pheromone receptors in moths mediate sex pheromone information, mutations causing the loss of function in these receptors may cause a deficit in pheromone-induced behavioural responses. The silkmoth, *Bombyx mori*, is considered as one of the best models to test this hypothesis and characterize the *in vivo* functional role of sex pheromone receptors in moths, because this species possesses the simplest possible pheromone system and is amenable to genetic manipulation. Female silkmoths emit two pheromone components (*E*, *Z*)-10,12-hexadecadienol (bombykol) and (*E*, *Z*)-10,12-hexadecadienal (bombykal), but only bombykol is sufficient to elicit full sexual behaviour including pheromone-source searching behaviour and copulation attempts by male silkmoths^20^,^21^,^22^,^23^. These pheromone components are detected by a pair of ORNs, each of which is highly specific to either bombykol or bombykal, in the pheromone-sensitive long sensillum trichodea on male antennae^21^. In the silkmoth, two sex pheromone receptors BmOR1 and BmOR3 are mutually exclusively expressed in a pair of ORNs in the pheromone-sensitive trichodeum sensillum and are highly specific to bombykol and bombykal, respectively^7^,^8^. Previously, we demonstrated that the artificial activation of BmOR1-expressing ORNs is sufficient to trigger pheromone-source searching behaviour in male silkmoths^24^,^25^. This finding suggests that BmOR1 is the sex pheromone receptor that mediates the pheromone response in male silkmoth. However, the causal relationship between BmOR1 function and pheromone responses has yet to be demonstrated.

In the present study, we established a BmOR1-knockout silkmoth line by employing TALEN-mediated genome editing technology. Electrophysiological responses of antennae and bombykol-sensitive ORNs were fully abolished in knockout male moths, whereas bombykal-sensitive ORNs in the same sensillum responded to bombykal in a dose-dependent manner. The loss of functional BmOR1 has no effect on the projections of bombykol-sensitive ORNs in the antennal lobe. BmOR1-knockout male moths never exhibited pheromone-source searching behaviour in response to bombykol or female silkmoths. Furthermore, the transgenic rescue of the *BmOR1* gene in bombykol-sensitive ORNs restored the behavioural responses of BmOR1-knockout males. These results demonstrate that BmOR1 is the receptor responsible for pheromone responses in the silkmoth; thus, providing the first direct evidence that a member of moth sex pheromone receptor actually mediates pheromone responses for sexual behaviour in *in vivo*.

## Results

### Generation of BmOR1-knockout silkmoths

**Figure 1:**
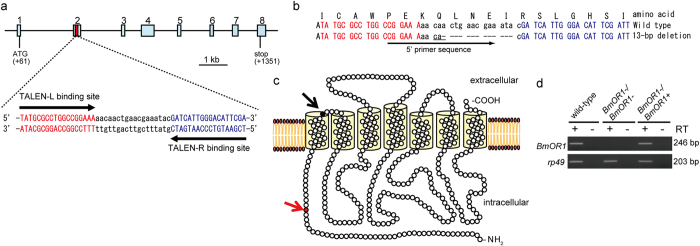
Generation of BmOR1-knockout silkmoths by using TALENs. (**a**) Schematic representation of the genomic structure of the *BmOR1* gene (top) and TALEN targeting sequences (bottom). Exons are indicated by light blue boxes and the start/stop codon locations are shown. TALENs were constructed to target sequences in the 2nd exon, as shown by the red box. Sequences of TALENs recognition sites are shown at the bottom of the genomic structure. (**b**) A TALEN-induced mutation allele used in this study. The wild-type sequence of *BmOR1* is shown at the top. The mutagenized *BmOR1* sequence is shown at the bottom. Deletions are indicated by the dashed line. Right and left TALEN recognition sequences are highlighted in red and blue characters, respectively. The black arrow under the sequences indicates the 5′ primer site used for RT-PCR amplification in (d). The 3′ primer was designed at 226 bp downstream of the 5′ primer sequence and is not shown in the figure. (**c**) Schematics of the predicted knockout BmOR1 protein. The predicted tertiary structure of BmOR1 protein is shown. The red arrow and amino acid residue indicate the position of a frame shift caused by the deletions, whereas the black arrow and circle indicate the position of the stop codon induced by the frame shift. (**d**) RT-PCR analysis of *BmOR1* gene expression. RT-PCR products by using RNA isolated from male antennae from wild-type individuals, mutation homozygous individuals (*BmOR1-/BmOR1*-), or mutation heterozygous individuals (*BmOR1-/BmOR1+*) were separated by electrophoresis. *rp49* was used as a positive control in the experiments. Plus and minus signs indicate that RT-PCR was performed with and without reverse transcriptase (RT), respectively.

To characterize the functional role of a sex pheromone receptor BmOR1 for the detection of bombykol and corresponding behavioural responses in *in vivo*, we generated BmOR1-knockout silkmoths using TALENs that were designed to target the 2nd exon of the *BmOR1* gene (Fig. 1a). We obtained various alleles that have a deletion, an insertion, or both deletion and insertion (Supplementary fig. 1, Supplementary table 1), and showed the availability of TALEN-mediated gene targeting in the silkmoth. From these genetic lines, we selected a line that had a 13-base deletion (Fig. 1b). This deletion caused a frame shift at the 40th amino acid residue of the BmOR1 protein and the introduction of a stop codon at the 1st extracellular loop region, resulting in the synthesis of a loss of function BmOR1 protein (Fig. 1c).

When the RT-PCR experiments were performed with a 5′ primer designed to anneal the deleted sequence, no amplification products were detected from the homozygous mutant (hereafter referred to as *BmOR1-/BmOR1+*) male antennae, whereas the products of the expected size were amplified from wild-type male antennae and also from the heterozygous mutant (hereafter referred to as *BmOR1-/BmOR1+ *) male antennae (Fig. 1d). Sequence analysis of the RT-PCR products amplified from *BmOR1-/BmOR1*- male antennae with another primer pair that spans the deletion region revealed that all transcripts have the same deletion as that of the genomic sequence, further supporting that this allele does not encode the mature BmOR1 protein. Notably, *BmOR1-/BmOR1*- individuals became adults in the same manner as their wild-type counterparts. Major morphological differences were not observed between *BmOR1-/BmOR1*- and wild-type moths. These observations indicated that the loss of BmOR1 does not affect the development and associated behaviour, such as the feeding behaviour, of silkmoths.

### Loss of BmOR1 does not affect projection patterns of bombykol-sensitive ORNs

**Figure 2:**
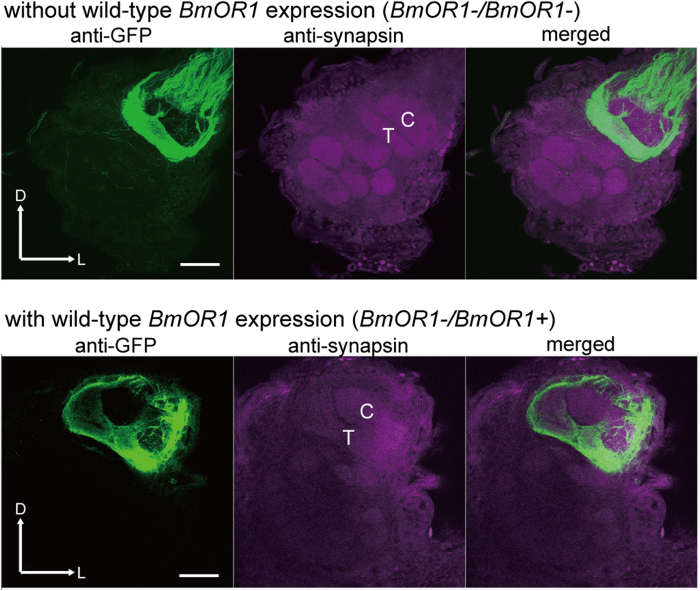
BmOR1-knockout does not affect the axonal projections of bombykol-sensitive ORNs. The axon terminals of bombykol-sensitive ORNs in the AL of *BmOR1-/BmOR1*- (top) or *BmOR1-/BmOR1+*  male moths (bottom) were visualized with GCaMP followed by anti-GFP immunostaining (green). Background staining was carried out with an anti-synapsin antibody to visualize neuropil structure (magenta). Representative confocal sections are shown. C: cumulus, T: toroid, D: dorsal, L: lateral. Scale bar: 50 μm.

To exclude the possibility that the loss of functional BmOR1 affects the projection patterns of bombykol-sensitive ORNs, we examined the axonal projection of these ORNs. Sex pheromone signals detected by ORNs are transmitted to a male-specific pheromone-processing structure called the macroglomerular complex (MGC) in the antennal lobe (AL), which is the first olfactory centre in insects^26^,^27^. BmOR1-expressing ORNs are reported to project to a single subdivision, named the toroid, of the MGC^24^,^28^,^29^. To examine the projection patterns of ORNs, we generated transgenic moths expressing a fluorescent protein GCaMP2 under the control of putative *BmOR1* promoter sequences by using the GAL4-UAS system^30^ (See Materials and Methods for details of a *BmOR1-GAL4* line). Through several rounds of crosses between *BmOR1-GAL4*, *UAS-GCaMP*, and BmOR1-knockout moths, we generated *BmOR1*-/*BmOR1*- male moths bearing both *BmOR1-GAL4* and *UAS-GCaMP* transgenes. As control experiments, we also generated *BmOR1*-/*BmOR1+* male moths, which express wild-type *BmOR1*, bearing both *BmOR1-GAL4* and *UAS-GCaMP* transgenes. GCaMP expression was only detected in the toroid of both *BmOR1*-/*BmOR1*- and *BmOR1*-/*BmOR1+ *AL (Fig. 2) as previously observed in moths with a wild-type background^24^,^31^. These results indicate that the loss of functional BmOR1 expression did not affect the projection patterns of BmOR1-expressing ORNs and suggest that BmOR1-knockout males develop proper neural connectivity between ORNs and olfactory neurons in the AL.

### BmOR1 is required for electrophysiological response to bombykol

**Figure 3:**
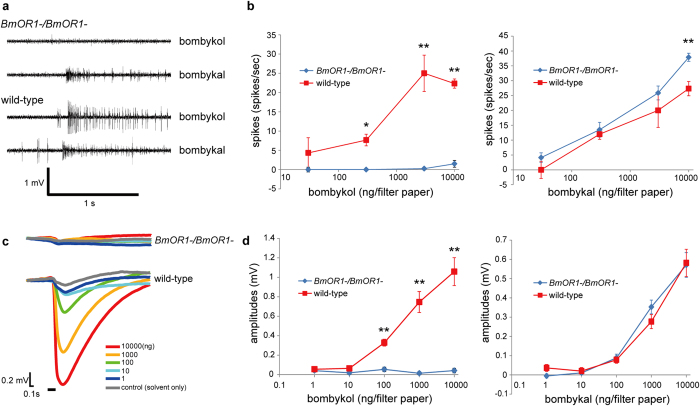
Electrophysiological responses of BmOR1-knockout male moths to bombykol. (**a**) Typical single sensillum recordings from the long sensillum trichodea of *BmOR1-/BmOR1*- and wild-type male moths to 10 μg bombykol or bombykal. The stimulus was applied for 1 s, as indicated by the solid line under the traces of the recordings. (**b**) Dose-dependent increases in the bombykol (left) or bombykal (right) -induced spike frequency of *BmOR1-/BmOR1*- (blue; n = 8) and wild-type (red; n = 3) male moths. Error bars represent ± SEM. Responses to bombykol and bombykal were recorded from the same sensilla. The asterisks indicate significant differences between groups (*p < 0.05, **p < 0.01) using the unpaired Student’s t-test for comparing pairs of data. (**c**) Typical EAG responses of *BmOR1-/BmOR1*- (top) and wild-type (bottom) male antennae to bombykol pulses of different odorant concentrations. The stimulus was applied for 200 ms, as indicated by the solid line under the trace. (**d**) Dose-dependent increases in bombykol (left) or bombykal (right) -induced peak EAG amplitudes of *BmOR1-/BmOR1*- (blue; n = 17) and wild-type (red = 5) male moths. Error bars represent ± SEM. The asterisks indicate significant differences between groups (**p < 0.01) using the unpaired Student’s t-test for comparing pairs of data.

Having shown the normal projection patterns of BmOR1-expressing ORNs in BmOR1-knockout silkmoths, we examined the electrophysiological properties of bombykol-sensitive ORNs in BmOR1-knockout males to bombykol by single sensillum recordings of long sensillum trichodeum. Spikes from two ORNs in this type of sensillum can be sorted by their amplitudes; the bombykol-sensitive ORN produces large amplitude spikes, while the bombykal-sensitive ORN produces small amplitude spikes (Fig. 3a, Supplementary fig. 2)^21^,^22^,^24^. Bombykol stimulation elicited no increase in the number of spikes from bombykol-sensitive ORNs of *BmOR1-/BmOR1*- male moths (Fig. 3a,b). In contrast, bombykol stimulations elicited a dose-dependent increase in the number of spikes from bombykol-sensitive ORNs in wild-type male moths (Fig. 3a,b). As control experiments, single sensillum responses to bombykal were recorded from the same sensilla. Bombykal evoked a clear dose-dependent increase in the number of spikes from bombykal-sensitive ORNs in both *BmOR1-/BmOR1*- and wild-type males (Fig. 3a,b). These results demonstrate that BmOR1 is required for the detection of bombykol, which is consistent with the results of previous heterologous experiments stating that BmOR1 is narrowly tuned to bombykol^8^. Notably, we detected spontaneous spikes with two distinct spike amplitudes in *BmOR1-/BmOR1*- males, showing that bombykol-sensitive ORNs are viable and capable of generating spikes without functional BmOR1 expression (Supplementary fig. 2), though the number of spontaneous spikes of bombykol-sensitive ORNs were largely reduced in *BmOR1-/BmOR1*- males (0.73 ± 0.29 spikes/s in wild-type, n = 22 and 0.15 ± 0.15 spikes/s in *BmOR1-/BmOR1*-, n = 13).

Whole-genome sequencing revealed the presence of 66 *OR* genes on the silkmoth genome^32^. Within these sequences, 5 *ORs*, including *BmOR1* and *BmOR3*, have been shown to be expressed in a male antennae-biased manner and have been included in the moth sex pheromone receptor clade in the phylogenetic tree of insect ORs^8^,^33^. Previous functional analyses that utilized the *Xenopus* oocyte expression system revealed that out of these 5 ORs only BmOR1 responds to bombykol, suggesting that BmOR1 is most likely the sole receptor for the detection of bombykol^8^. If this is the case, we reasoned that the loss of BmOR1 would completely abolish the electrical responsiveness of the male antennae to bombykol. To confirm this hypothesis, we measured the sum of the electrical responses of the ORNs from the whole antenna by electroantennogram (EAG) recordings. Upon stimulation with bombykol, the antennae of *BmOR1*-/*BmOR1*- did not show any responses, whereas those of wild-type males responded dose dependently (Fig. 3c,d). As expected from single sensillum responses to bombykal, bombykal evoked a dose-dependent response in both *BmOR1*-/*BmOR1*- and wild-type male antennae (Fig. 3d). Although we cannot completely exclude the possibility that very weak responses cannot be detected by relatively insensitive EAG recording, our results strongly support that BmOR1 is the sole receptor for the detection of bombykol in male silkmoth antennae.

### BmOR1 is required for the initiation of pheromone-source searching behaviour

**Figure 4:**
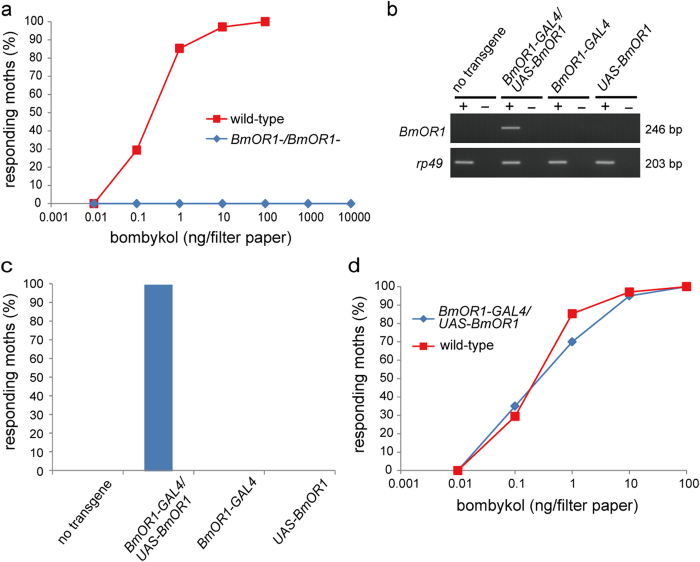
BmOR1-knockout completely abolishes the behavioural responses of male moths to bombykol. (**a**) Behavioural response percentages of *BmOR1-/BmOR1*- (blue, n = 14) and wild-type (red, n = 34) male moths to different concentrations of bombykol were plotted. None of the *BmOR1-/BmOR1*- male moths displayed behavioural responses to any of the bombykol concentrations. (**b**) Transgenic rescue of the *BmOR1* gene. RT-PCR analysis of *BmOR1* gene expression in the antennae of *BmOR1-/BmOR1*- male moths bearing no transgene, both *BmOR1-GAL4* and *UAS-BmOR1, BmOR1-GAL4*, or *UAS-BmOR1*. The primer pair in Fig. 1d that amplified only wild-type *BmOR1* transcripts was used. *rp49* was used as a positive control in the experiments. Plus and minus signs indicate that RT-PCR was performed with and without reverse transcriptase, respectively. (**c**) Behavioural response percentages of *BmOR1-/BmOR1*- male moths with the indicated transgene(s). The moths were exposed to 100 ng bombykol. The number of samples was as follows: no transgene (n = 14), *BmOR1-GAL4*/*UAS-BmOR1* (n = 20), *BmOR1-GAL4* (n = 12), and *UAS-BmOR1* (n = 12). (**d**) Dose-dependent increase in the percentages of *BmOR1-/BmOR1*- male moths with *BmOR1-GAL4* and *UAS-BmOR1* (blue, n = 20) and wild-type male moths (red, n = 34) that responded to bombykol.

Since knockout of the *BmOR1* gene completely abolished antennal responses to bombykol, the loss of behavioural responses to bombykol would be expected. Thus, we examined how the loss of functional BmOR1 expression affected the initiation of pheromone-source searching behaviour. Upon stimulation with bombykol, *BmOR1*-/*BmOR1*- males showed neither wing flapping behaviour nor walking behaviour, which are the characteristics of pheromone-source searching behaviour in the silkmoth^23^,^34^, even at the highest dose of bombykol, which was 2-orders higher than that required to elicit the behavioural responses in all wild-type males (Fig. 4a, Supplementary video 1). These results indicate that BmOR1 is responsible for the detection of bombykol, triggering male silkmoth behavioural responses. Since bombykol is believed to be the sole stimulant that elicits the pheromone-source searching behaviour of male moths, we assumed that *BmOR1*-/*BmOR1*- male moths also lose the ability to recognize female silkmoths. Indeed, when exposed to a female moth in a wind tunnel, *BmOR1*-/*BmOR1*- male moths never exhibited orientation behaviour toward female moths, and did not perform wing flapping or walking behaviour, whereas wild-type displayed pheromone-source searching behaviour (Supplementary video 2). These results demonstrate that specific interaction of BmOR1 and bombykol is essential for the initiation of pheromone-source searching behaviour, and thus for recognizing the presence of conspecific females.

Finally, to further confirm that the loss of behavioural responses is caused by the loss of BmOR1 function, and to exclude the possibility of the off-target effect of the TALENs used in this study, we generated a transgenic rescue line of BmOR1 by using the GAL4-UAS system. For this aim, we established an effector line that possesses *UAS-BmOR1* transgene that is designed to express wild-type *BmOR1* under the control of *UAS*. Intact *BmOR1* mRNA expression was restored in the male antennae of *BmOR1*-/*BmOR1*- bearing both *BmOR1-GAL4* and *UAS-BmOR1* transgenes (Fig. 4b). Upon stimulation with bombykol, male *BmOR1*-/*BmOR1*- moths bearing both *BmOR1-GAL4* and *UAS-BmOR1* transgenes exhibited wing flapping behaviour with almost the same sensitivity as observed for wild-type male moths (Fig. 4c,d), demonstrating the presence of a causal relationship between BmOR1 expression in bombykol-sensitive ORNs and the initiation of pheromone-source searching behaviour.

## Discussion

Since the discovery of *BmOR1* as the first sex pheromone receptor gene from moth species in 2004, functional characterization of sex pheromone receptor genes have been reported in more than 10 moth species^7^,^8^,^9^,^10^,^11^,^12^,^13^,^14^,^15^,^16^,^17^,^18^,^19^. Despite advancement of understanding of ligand-receptor relationship, there has been no demonstration of functional role of these receptors *in vivo*. Here, by employing TALENs-mediated gene targeting strategy combined with physiological and behavioural analyses, we provide the first direct evidence that a member of the moth sex pheromone receptors mediates sex pheromone information to elicit pheromone-source searching behaviour *in vivo*.

In the present study, we showed that the loss of functional BmOR1 expression completely abolished both the behavioural and electrophysiological responses of male moths to the female silkmoth sex pheromone bombykol, demonstrating that BmOR1 is necessary for the recognition of bombykol in *in vivo*. We have previously shown that the activation of BmOR1-expressing ORNs is sufficient to trigger full sexual behaviour in male silkmoths^24^. These findings combined with those of the present study indicate that the activation of BmOR1-expressing ORNs evoked by the interaction of BmOR1 and bombykol is necessary and sufficient for triggering sexual behaviour in male silkmoths. Thus, BmOR1 is the sole receptor that mediates the bombykol response in the silkmoth.

Unlike the silkmoth, most moth species utilize blends of multiple pheromone components^2^, with species-specific blend ratios being important for the initiation of pheromone-searching behaviour and localising female moths^35^. The males of these moth species, generally, possess multiple sex pheromone receptors, each of which specifically responds to a single pheromone component or, in some cases, to several sex pheromone components of the corresponding species^10^,^11^,^12^,^13^,^14^,^15^,^16^,^17^,^18^,^19^. Therefore, blend information should be represented by the activities of different types of ORNs expressing distinct sex pheromone receptors at a peripheral level. This implies that complex processing will occur at the level of the AL and higher centres of the brain to extract blend information. The gene knockout strategy used in this study is expected to help clarify the functional roles of each sex pheromone receptor and how pheromone blend information is coded by moth brains.

Although our results clearly demonstrated that BmOR1 functions as a gatekeeper for the initiation of pheromone-source searching behaviour in male silkmoths, other molecular components, such as the pheromone-binding protein^36^ and sensory neuron membrane protein^37^, have also been reported to contribute to the specificity and sensitivity of pheromone detection by ORNs^38^,^39^. We exemplified that TALENs-mediated gene targeting may be applied effectively to identify the *in vivo* functions of olfactory genes. The high efficiency of TALENs-mediated genome editing in the silkmoth^40^ provides a useful platform to characterize the functions of these genes *in vivo*.

We found that the axons of bombykol-sensitive ORNs in BmOR1-knockout males were correctly projected into the toroid in the AL. We previously reported that the additional expression of a diamondback moth sex pheromone receptor PxOR1 in bombykol-sensitive ORNs does not change the projection pattern of these ORNs in the AL^24^. Thus, sex pheromone receptors are not likely to be involved in targeting the ORNs axon terminals in moths. Similar observations have been reported in the fruit fly olfactory system, in which ORNs tuned to general odorants or a volatile pheromone, *cis*-vaccenyl acetate (cVA), projected to a fixed glomerulus, regardless of *OR* gene expression^41^,^42^. Therefore, unlike the vertebrate olfactory system, in which the expressed OR instructs the projection pattern of ORNs^43^, insect ORs are not likely to be generally involved in the axonal projections of ORNs.

In conclusion, we demonstrate that the knockout of the sex pheromone receptor BmOR1 results in the loss of both physiological and behavioural responses to the sex pheromone bombykol, without the modification of ORN input to the brain. Our results provide the first direct evidence that a member of the moth sex pheromone receptors actually functions as the “sex pheromone receptor” rather than as ORs specific to sex pheromone substances. Comparative analysis of “sex pheromone receptors” in various moth species will help to unravel the evolution of the molecular function that is causally related to pheromone preference in moths.

## Methods

### Animals and chemicals

The w1-pnd strain of *Bombyx mori*, which is non-diapausing and has non-pigmented eggs and eyes, was used for the generation of BmOR1-knockout moths. Larvae were reared on an artificial diet (Nihon Nosan Kogyo, Yokohama, Japan) at 25 °C on a 16:8 h (light/dark) photoperiod cycle. Synthetic bombykol and bombykal were provided by Dr. S. Matsuyama from the University of Tsukuba, Japan.

### Construction of TALEN expression vectors

TALEN expression vectors were constructed as described previously using a two-step Golden Gate assembly method^44^. Briefly, each nucleotide-specific monomer sequence with ligation adaptors was generated using PCR. Then, the appropriate monomers were assembled into hexamer by a first Golden Gate reaction. The assembled hexamers were amplified by PCR and subsequently assembled into the appropriate TALE nuclease backbone vector^44^ by a second Golden Gate reaction, to yield fully assembled, sequence-specific TALENs.

### Synthesis of RNA for injection

The TALEN vectors were purified by using a Qiagen Hispeed plasmid midi kit (Qiagen, Hilden, Germany), digested with *Not*I restriction enzyme, and used as templates for *in vitro* transcription with a mMESSAGE mMACHINE T7 Ultra Transcription kit (Ambion, Austin, USA) according to the manufacturer’s instruction. RNA was purified by LiCl precipitation followed by 4 washes with 70% ethanol. RNA of left and right TALENs were dissolved at a concentration of 0.2 μg/μl each in 0.5 mM phosphate buffer (pH 7.0) containing 5 mM KCl. RNA solution was injected into preblastoderm stage embryos, as previously described^45^,^46^.

### Screening of mutagenized moths

G_1_ eggs were obtained by the sibling mating of G_0_ adults. Genomic DNA of G_1_ eggs from different broods was extracted separately using a DNeasy Blood & Tissue Kit (Qiagen). The region surrounding the target site was amplified by PCR using genomic DNA as the template with the primers (sense: 5′-ACCAAGGTCAGTTTCGGCTATAAAAG-3′ and antisense: 5′-CACAAGTAACCAAATTCATCATCACAG-3′). The PCR products were directly sequenced using an ABI3700 DNA analyser (Applied Biosystems, Foster City, CA, USA). G_1_ broods that showed overlapping sequencing patterns with the target sequence were reared to adults. After being crossed with wild-type adults, the genomic DNA of G_1_ moths was extracted, PCR amplified, and sequenced, as described in this section, to identify mutagenized individuals. In the silkmoth, males are the homogametic sex with *ZZ* sex chromosomes, whereas the females are the heterogametic sex with *ZW* sex chromosomes. Since *BmOR1* is located on the *Z* sex chromosome, we crossed *BmOR1*-/*W* females with *BmOR1*-/*BmOR1+* males to obtain *BmOR1*-/*BmOR1*- males in next generation.

### Generation of transgenic silkmoths

For the *UAS-BmOR1* construct, the entire protein-coding sequence of *BmOR1* was subcloned immediately downstream from the *UAS* of pBacMCS-UAS^47^. The resultant vector was used to generate a *UAS-BmOR1* line. Because transgene of the previously established *BmOR1-GAL4* line^23^ was found to be located on *Z* chromosome as with *BmOR1*, we generated a new *BmOR1-GAL4* line. For the *BmOR1-GAL4* construct, approximately 2.0 kb DNA fragments immediately upstream from the initiation codon of *BmOR1* were amplified using the PCR from the w1-pnd silkmoth genome DNA, and subcloned into the *Asc*I-*Bam*HI site of pBacMCS-GAL4 (ref. 48). The resultant vector was used to generate a *BmOR1-GAL4* line used in this study. Transgenic silkmoths were generated using the *piggy*Bac-mediated germ-line transformation method, as described previously^45^,^49^.

### Reverse-transcription (RT)-PCR

Total RNA was extracted from the antennae of male moths at 1–5 days after eclosion using TRIzol reagent (Invitrogen, Carlsbad, CA, USA), treated with DNase I, and reprecipitated. RNA was reverse transcribed using an oligo(dT) adaptor primer (Takara-Bio, Otsu, Japan) and AMV reverse transcriptase (Takara-Bio) at 42 °C for 35 min. The cDNA of *BmOR1* and *B*. *mori ribosomal protein49* (rp49) was amplified using Ex *Taq* DNA polymerase (Takara-Bio) and the primer pairs for *BmOR1* (5′-CGGAAAAACAACTGAACGAA-3′ and 5′-CCGTTATGAAGCGACCAGTT-3′) and *B*. *mori rp49* (5′-CAGGCGGTTCAAGGGTCAATAC-3′ and 5′-TGCTGGGCTCTTTCCACGA-3′), with thermal cycling at 95 °C for 2 min, followed by 30 cycles at 95 °C for 30 s, 50 °C for 15 s, and 72 °C for 20 s, and 72 °C for 5 min. Equal amounts of the PCR products were separated by electrophoresis on 2.0% agarose gels. No PCR products were produced when reverse transcriptase was excluded during reverse transcription, and sequence analysis confirmed the identity of the cDNA products. For confirmation of the deletion sequence in *BmOR1-/BmOR1*- male antennal transcripts, the following primer pair was used (sense: 5′-ACCAAGGTCAGTTTCGGCTATAAAAG-3′ and antisense: 5′-CACAAGTAACCAAATTCATCATCACAG-3′).

### Immunohistochemistry

Immunohistochemical staining of moth brains was carried out as described previously with minor modifications^24^. Anti-synapsin antibody (developmental studies of the hybridoma bank) and Alexa546-conjugated goat anti mouse IgG secondary antibody (Invitrogen) were used for neuropil staining in this study, rather than anti-synaptotagmin and Cy3-conjugated donkey anti mouse IgG secondary antibody, which were used in the previous study. Confocal images were captured using a LSM510 confocal system (Carl Zeiss, Jena, Germany).

### Electroantennogram recordings

Electroantennogram recordings were essentially performed as described previously^31^. In brief, glass micropipettes with silver chloride wire were filled with Ringer’s solution and inserted into the distal and proximal ends of a male antenna mounted in a portable chamber. To protect them from drying, the cut ends of the antenna were covered by gel (SPECTRA 360; Parker Laboratories, Fairfield, NJ, USA). A glass cartridge (5 mm inner diameter) was prepared for stimulation by inserting a piece of filter paper (1 × 2 cm) containing 5 μl of the dissolved odorant solution or *n*-hexane as the control. A charcoal-purified airstream was passed through the glass pipette and directed on the antenna. The EAG responses were amplified (Multiclamp 700b; Molecular Devises, Sunnyvale, CA, USA), low-pass filtered at 0.01 kHz and digitized at 10 kHz (USB-X series; National Instruments, Austin, TX, USA). Data was analysed by using a custom written program (MATLAB; Mathworks, Natick, MA, USA).

### Single sensillum recordings

Single sensillum recordings were carried out as described previously^24^. In brief, the moth was immobilized on a plastic plate under an Olympus BX50 ( ×500) microscope (Olympus, Tokyo, Japan), and the antenna was stabilized by dental wax. Spikes were recorded by inserting an electrolytically sharpened tungsten electrode (diameter 0.25 mm, tip approximately 1 μm) into the bases of the long sensillum trichodea on the male antennae. A reference electrode was inserted in the body of the moth. Pieces of filter paper containing different odours were set into the glass pipette. A charcoal-purified airstream was passed through the glass pipette and directed on the antenna. The airflow rate was adjusted to 1 l/min. The response was amplified by an amplifier (MEZ-8300, Nihonkoden, Tokyo, Japan) and electrophysiological data were obtained by using Digidata1322 (Axon Instruments, Union City, CA, USA). The responses were quantified by counting spikes during 1 s following the onset of the stimulus and subtracting the number of mean spontaneous spikes/s in a 1-s time window prior to stimulation.

### Behavioural experiments

Behavioural responses to bombykol were examined as described previously^24^. Male silkmoths were used within 1–7 days after eclosion. The moths (up to 5 per experiment) were placed in a translucent cylindrical acrylic closed box (15 cm in diameter and 6.5 cm in height). An air-puff stimulus was used to spread odorants into the box through a 2-mm-diameter hole in the middle of the lid with a Pasteur pipette containing a piece of filter paper with the odorant. A charcoal-purified airstream (1.4 l/min) was passed through a Pasteur pipette and directed into the box. Pulsed odorant stimulation (200 ms duration) was produced by controlling a three-way solenoid valve by a custom written program (LabVIEW; National instruments). The odorants were dissolved in n-hexane, and applied to a piece of filter paper (1 × 2 cm). The moths were exposed to increasing concentrations of bombykol (0.01, 0.1, 1, 10, 100, 1000, and 10000 ng) at 1-min intervals. The air and odorant were removed through an exhaust tube attached to the side of the box 10 s after each puff stimulus. Wing flapping within 10 s of the stimulation that lasted for more than 10 s was counted as a response.

### Statistical analysis

The error bars represent SEMs. To assess statistical significance between wild-type and BmOR1-knockout moths, we used the unpaired Student’s t-test for comparing pairs of data, using Microsoft Excel 2010 and a commercial macroprogram (Statcel version 3, Seiun-sya, Japan).

## Additional Information

**How to cite this article**: Sakurai, T. *et al*. Targeted disruption of a single sex pheromone receptor gene completely abolishes *in vivo* pheromone response in the silkmoth. *Sci. Rep*. **5**, 11001; doi: 10.1038/srep11001 (2015).

## Supplementary Material

Supplementary Information

Supplementary Video 1

Supplementary Video 2
