# Supplementary material for: Targeted disruption of a single sex pheromone receptor gene completely abolishes in vivo pheromone response in the silkmoth
Source: Sci Rep. 2015 Jun 5;5:11001. doi: 10.1038/srep11001 (PMC4457163; doi:10.1038/srep11001)
Supplement: Supplementary Information [file srep11001-s1.pdf]

Supplementary information

Targeted disruption of a single sex pheromone receptor gene completely abolishes *in vivo* pheromone response in the silkmoth

Takeshi Sakurai, Hidefumi Mitsuno, Akihisa Mikami, Keiro Uchino, Masashi Tabuchi, Feng Zhang, Hideki Sezutsu, Ryohei Kanzaki

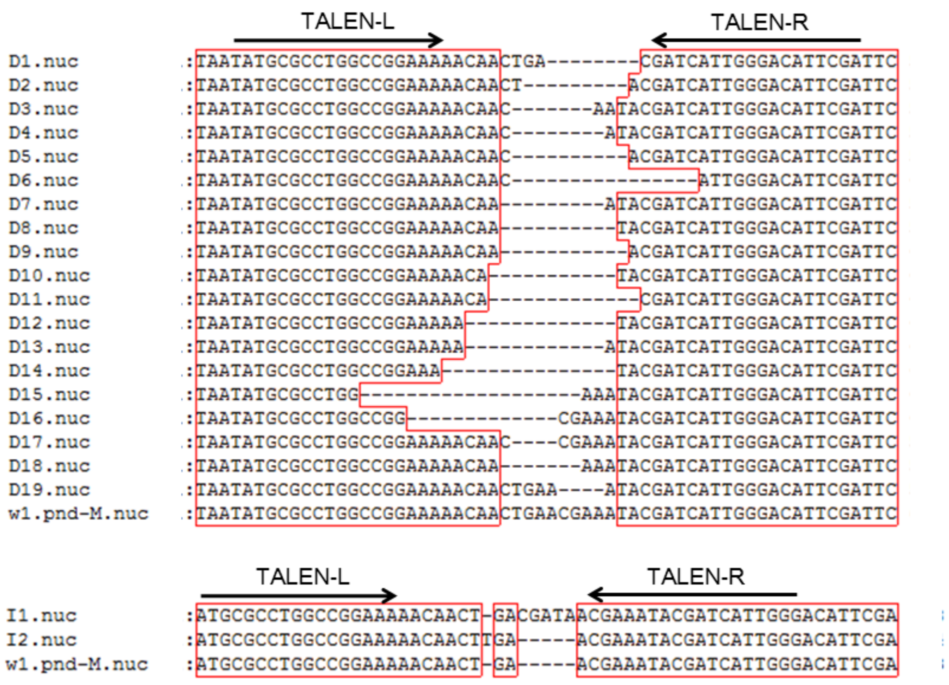

Supplementary fig. 1. Sequence comparison of *BmOR1* alleles produced by TALENs. TALEN target sites are indicated by black arrows. Allele names starting with “D” indicate deletion alleles, whereas those starting with “I” indicate insertion alleles. w1.pnd-M indicates the wild-type DNA sequence. Mutants with D11 allele were used in this study.

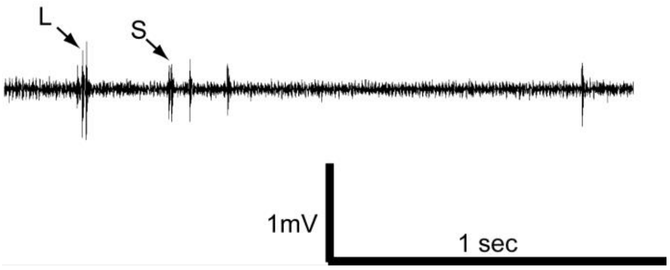

Supplementary fig. 2. Typical single sensillum recording trace of sensillum trichodea of BmOR1-knockout males in the absence of pheromone stimulations. Spontaneous spikes with two different amplitudes were generated. Large (L) and small (S) amplitude spikes were from bombykol- and bombykal-sensitive ORNs, respectively.

Supplementary Table 1

Efficiency of TALEN-mediated mutagenesis of the *BmOR1* gene

# of G1 brood	# of mutated	Ratio of mutation	# of G1 adults	# of mutated	Ratio of mutants
15	12	80.0%	169	112	66.3%

Caption for Supplementary Videos

Supplementary Video 1. Representative behavioural responses of BmOR1-knockout males and wild-type males to bombykol stimulation. BmOR1-knockout (*BmOR1-/BmOR1-*) (top two moths) males did not display wing flapping or walking behaviour, which are the criteria for the display of pheromone-source searching behaviour by silkmoths, following stimulation with 1,000 ng bombykol (second stimulus). Wild-type males (bottom two moths) vigorously exhibited pheromone source searching behaviour to 1,000 ng bombykol stimulation (second stimulus). Hexane (first stimulation) is a solvent of bombykol that was used for negative control stimulation. LED lights indicate the timing of stimulation (See Behavioural experiments in Methods for details of the way of pheromone stimulation).

Supplementary Video 2. Representative behavioural responses of BmOR1-knockout males and wild-type males to a female silkmoth. Even when exposed to a female moth, BmOR1-knockout (*BmOR1-/BmOR1-*) (top and bottom moths) males did not display pheromone-source searching behaviour, whereas a wild-type male (middle moth) immediately initiated pheromone source searching behaviour, located a female, and copulated with it. Male moths were placed downwind of a female moth with a wind velocity of 0.4m/s in a wind tunnel (180 cm long, 90 cm wide, and 30 cm high).
